# The role of spoken language in cardiovascular health inequalities: a cross-sectional study of people with non-English language preference

**DOI:** 10.3399/bjgpopen17X101241

**Published:** 2017-11-29

**Authors:** Alex Mackay, Mark Ashworth, Patrick White

**Affiliations:** 1 Academic Fellow, School of Population Health and Environmental Sciences, King’s College London, Guy’s Campus, London, UK; 2 Reader in Primary Care, School of Population Health and Environmental Sciences, King’s College London, Guy’s Campus, London, UK; 3 Reader in Primary Care, School of Population Health and Environmental Sciences, King’s College London, Guy’s Campus, London, UK

**Keywords:** cardiovascular health inequalities, ethnicity, language preference

## Abstract

**Background:**

Socioeconomic and ethnic factors are established determinants of cardiovascular health inequalities. The role of low proficiency in the majority language as a mediator of these inequalities is uncertain.

**Aim:**

This study aimed to investigate the association between non-English language preference and cardiovascular health inequalities in a community in London.

**Design & setting:**

Retrospective, cross-sectional analysis of anonymised patient-level data collected from general practices in Lambeth, south London.

**Method:**

Cardiovascular disease prevalence, monitoring, and risk-identification data were compared between non-English and English language groups using multiple logistic regression.

**Results:**

Of the total number of patients registered at the 49 participating practices, 302 404 (83%) patients were aged ≥18 years. Preferred language was recorded by 69.4%: English 53.6%, Portuguese 3.2%, Spanish 2.6%, French 1.6%, Polish 1.4%, Somali 0.5%, and others 7.1%; 30.6% had no record of language preference. The non-English language preference group had a greater likelihood of coronary heart disease ([CHD], odds ratio [OR] 1.18, 95% confidence interval [CI] = 1.03 to 1.34); diabetes mellitus ([DM], OR = 1.33, 95% CI = 1.23 to 1.43); obesity (OR = 1.08, 95% CI = 1.04 to 1.13); and smoking (OR = 1.18, 95% CI = 1.14 to 1.21), but no difference in the prevalence of hypertension or stroke. Cardiovascular monitoring was not less intense in this group. Portuguese-speakers (the largest non-English language preference group) had a greater likelihood of hypertension (OR = 1.43, 95% CI = 1.30 to 1.57); DM (OR = 1.74, 95% CI = 1.50 to 2.02); stroke (OR = 1.40, 95% CI = 1.08 to 1.81); obesity (OR = 1.53, 95% CI = 1.36 to 1.73); and smoking (OR = 1.13, 95% CI = 1.02 to 1.25).

**Conclusion:**

The non-English language preference group was associated with a greater risk of some aspects of cardiovascular disease than the English language preference group, probably reflecting shared cultural and behavioural risk. Non-English language preference was not associated with lower rates of cardiovascular monitoring, providing some evidence of equitable primary care access in this group.

## How this fits in

Ethnicity is known to be an important determinant of cardiovascular disease. This study shows that, independent of ethnicity, non-English language preference (in this case, mainly Portuguese language preference) is also a risk factor for cardiovascular disease. Language preference is likely to define a social group with shared cultural and behavioural cardiovascular risk factors.

## Introduction

Health inequalities in cardiovascular disease, DM, and smoking are associated with ethnicity.^1^ The relationship between health inequality and ethnicity has been recognised as a global health priority.^2^ In the UK, the Marmot Review, an influential body of work on the socioeconomic determinants of health, was criticised for overlooking the significance of ethnicity.^3,4 ^In UK primary care, cardiovascular ethnic health inequalities were found to have persisted even as socioeconomic health inequalities decreased, following the introduction to general practice of a pay-per-performance system in 2004.^5^ Minority ethnic groups have been linked with disparities in prescribing for cardiovascular disease.^6,7^ The determinants of worse health outcomes linked to ethnicity are likely to be multifactorial, with evidence for socioeconomic, genetic, and cultural factors.^1^ Low proficiency in the majority language is an established risk factor for health inequalities among migrants in the US.^8,9^ Interpreting services in the UK have been underused, and only 65% of people with low proficiency in English have reported good health compared to 80% of those whose main language was English.^10,11^The challenge of communication is a theme that has recurred in the analysis of the health needs of minority groups with low proficiency in the majority language.^9^ The impact of low English proficiency on health among migrants in a UK setting has not been investigated before. Failure to acquire the local language may affect the way migrants interact with health services and may contribute to cardiovascular health inequalities. This failure may also be the result of poor health limiting migrants' opportunities to improve their English language skills.^11^

The London Borough of Lambeth, the setting of this research, has been in the top 10% of UK local authority areas for number of international migrants from 1951 to 2011.^12^ It has the highest proportion in the UK of Portuguese people, South Americans, mixed-race white and black African people, people from non-Caribbean and non-African black backgrounds, and people from multiple mixed ethnic backgrounds.^12^ Social deprivation according to the Index of Multiple Deprivation (IMD 2010) places Lambeth as the 5th most deprived out of 31 boroughs in London.^12^ The largest non-English language group in Lambeth is Portuguese. In the 2011 census, 9897 (3.4%) of the 290 080 people in Lambeth described themselves as Portuguese-speakers.^13 ^This group included individuals born in mainland Portugal, Madeira, and former Portuguese colonies in Latin America, Africa, and East Timor. Linguistic and cultural barriers, lack of information, and poor access have been identified as health priorities of the local Portuguese-speaking population.^14 ^Language barriers have been shown to contribute to educational inequalities in the Portuguese community.^15^ This study aimed to determine whether this language-based approach had a role in assessing health inequalities. More specifically, it aimed to determine if preference for a non-majority language was associated with cardiovascular health risks in terms of monitoring, risk factors, and diagnosis.

## Method

### Design, setting, and sample

This was a cross-sectional, population-based study set in Lambeth in inner south London in 2012. Anonymised data were obtained from Lambeth DataNet, a primary care database for Lambeth general practices. Patients registered at 49 of the 50 general practices in the borough were eligible; the remaining practice had incompatible clinical software. Patient inclusion was based on the location of the general practice where the patient was registered and not on the location of the patient's residence. The data extracted included demographic details, self-reported ethnic coding, language spoken, country of birth, cardiovascular diagnosis, cardiovascular risk factors, and cardiovascular monitoring.^16,17^ Data were recorded at patient registration. Practice staff were further encouraged to complete missing data either at reception or during consultations through the apperance of screen prompts. Patients aged <18 years were excluded because their communication with primary care services may have been mediated by a parent or carer.

### Language groups and patient characteristics

Patients’ language preferences were recorded as a free text entry without prompts. This record was interpreted as an indication of the patient’s preference for the language of communication with health services. It was assumed that the patient preferred not to speak in the majority language of the service, English, and that this preference reflected lower proficiency in English. The non-English language preference groups with the highest frequencies were identified and compared to the English language preference group with respect to cardiovascular diagnosis, cardiovascular risk factors, and cardiovascular monitoring. In a separate analysis, the non-English language preference groups, as well as all those who did not declare a language preference, were compared with the English language preference group. Ethnicity was defined using the categories of the 2011 census:^18^ white (British or mixed British, Irish, other white background); black or black British (African, Caribbean, or other black); Asian or Asian British (Bangladeshi or British Bangladeshi, Indian, or British Indian, other Asian background, Pakistani or British Pakistani); mixed (other mixed, white and Asian, white and black African, and white and black Caribbean); other ethnic (Chinese, other); unknown; or not stated. Age, sex, and country of birth were included in the analysis. Relative social deprivation was measured with the IMD 2010, calculated for each individual patient. IMD score is based on national census and local authority data, and reflects deprivation specific to a geographical area.^19^ Groups that preferred languages spoken by <0.5% of the population were excluded from the comparative analysis. This arbitrary cut-off was applied to promote clarity in the presentation of the findings.

### Cardiovascular health indicators

Cardiovascular diagnoses, risk factors, and monitoring criteria were derived from Quality and Outcomes Framework (QOF) data for the year 2011–2012. QOF is a pay-for-performance system, introduced in England in 2004, which is based on the achievement of performance indicators relating to clinical and organisational targets.^20^ Data analysis was based on all registered patients. The QOF process of ‘exception reporting’ was not included in the analysis. The cardiovascular diagnoses selected for analysis were the four long-term conditions; namely, CHD, hypertension, DM, and stroke. Risk factors analysed were current smoking status; blood pressure (BP) over target (BP >150/90 mmHg); total cholesterol over target (total cholesterol >5mmol/l); HbA1c over target (HbA1c >48 mmol/mol); and body mass index (BMI) >30 kg/m^2^. All BMIs >100 were assumed to be erroneous and excluded. Monitoring was evaluated by reference to whether or not there was a record of blood pressure within 9 months of the date of the survey, and of smoking cessation, serum cholesterol level, and HbA1c within 15 months of the survey.

### Statistical analysis

The demographic details of the English language preference group and all other groups were compared. Means for age and IMD scores were compared using independent *t*-tests. Sex proportions were compared using χ^2^ tests. The frequencies of the most numerous ethnicities and countries of birth for the language groups were listed as percentages. Associations between preferred language and patients’ cardiovascular diagnosis, risk factors (at the most recent measurement), and monitoring were assessed using multiple logistic regression to generate ORs with the English language preference group as the reference group. Adjustment in the regression was made for age, sex, ethnicity, social deprivation, and practice clustering. Statistical analysis was carried out using IBM-SPSS and Stata.

## Results

Of the 366 283 patients registered with the 49 included practices, 302 404 (83%) were aged ≥18 years and formed this study's sample. One hundred and three spoken languages were reported. Table 1 shows the distribution of the six languages spoken by ≥0.5% of the local population. The 97 other languages were named by 19 380 (6%) patients. All 103 languages were included in the analysis of data from the non-English language preference group. The demographic characteristics of the language groups are described in Table 1. The non-English language preference groups were significantly younger and significantly more deprived than the English language preference group. Fewer than half of the sample patients were born in the UK. Almost half of the non-English language preference group described their ethnicity as 'other white', a category comprising 67% of all responders and including groups from origins as diverse as eastern Europe and Latin America.

**Table 1.** Demographic characteristics compared between English language preference and other language preference groups using means (*t*-tests) and sex proportions (χ^2^).

Language preference	Mean age, years (SD)	Males, %	Mean IMD score (SD)	Ethnic groups, % (two most frequent and ‘Not recorded’)	Country of birth, % (UK, most frequent, and ‘Not recorded’)
English *n* = 161 938	41.4 (16.0)	47.9	30.1 (8.4)	British or mixed British, 44.8; Other white, 17.0; Not recorded, 16.5	UK, 47.1; Jamaica, 4.1; Not recorded, 22.6
Non-English *n* = 47 790	38.9 (13.5)^a^	48.2^a^	32.1 (8.4)^a^	Other white, 47.3; African, 13.9; Not recorded, 22.5	UK, 1; Portugal, 9.5; Poland, 7.5; Not recorded, 21.4
Portuguese *n* = 9862	38.9 (12.7)^a^	49.3^a^	33 (7.5)^a^	Other white, 66.6; Other, 8.2; Not recorded, 11.3	UK, 0.5; Portugal, 46.4; Brazil, 24.5; Not recorded, 19.6
Spanish *n* = 7882	38.8 (12.3)^a^	47.2^a^	33.2 (7.9)^a^	Other white, 50.1; Other, 26.5; Not recorded, 14.0	UK, 0.7; Colombia, 23.4; Spain, 20.7; Not recorded, 18.9
French *n* = 4968	36.5 (11.0)^a^	48.5^a^	32.1 (8.3)^a^	African, 37.8; Other white, 36.0; Not recorded, 13.5	UK, 0.8; France, 39.9; Ivory Coast, 11.7; Not recorded, 21.1
Polish *n* = 4311	35.6 (11.1)^a^	45.0^a^	31 (7.9)^a^	Other white, 90.3; British or mixed British, 5.7; Not recorded, 2.2	UK, 0.2; Poland, 81.9; Portugal, 0.3; Not recorded, 17.0
Somali *n* = 1567	39.3 (13.6)^a^	43.7^a^	34.6 (7.8)^a^	African, 75.9; Other black, 16.5; Not recorded, 20.9	UK, 0.5; Somalia, 73.1; Not recorded, 25
Other *n* = 19 380	40.3 (14.9)^a^	49.2^a^	31.3 (7.9)^a^	Other white, 33.7; African, 15.5; Not recorded, 38.6	UK, 1.7; Italy, 9.9; Pakistan, 4.4; Not recorded, 24.1
Not recorded*n* = 92 676	42.2 (14.9)^a^	58.4^a^	30.8 (8.7)^a^	Unknown, 50.3; British or mixed British, 13.6; Not recorded, 13.4	UK, 1.5; Ghana, 0.7; Not recorded, 90.9

^a^ *P* <0.01 in comparison with English language preference groups.

IMD = Index of Multiple Deprivation, 2010. Not recorded = no data were received for patients in this category. SD = standard deviation.

### Cardiovascular long-term conditions

The associations between cardiovascular diagnoses and language preference groups adjusted for age, sex, socioeconomic deprivation, ethnicity, and practice clustering are reported in Table 2. The non-English language preference group as a whole had higher prevalences of CHD and DM. Hypertension, DM, and stroke were more prevalent among the Portuguese language preference group.

**Table 2.** Association between cardiovascular diagnosis and language preference (adjusted^a^ ORs with 95% Cs).

	Hypertension	CHD	DM	Stroke
	OR (95% CI)	OR (95% CI)	OR (95% CI)	OR (95% CI)
**English ** *n* = 161 938	1	1	1	1
**Non-English ** *n* = 47 790	0.98 (0.92 to 1.03)	1.18 (1.03 to 1.34)^b^	1.33 (1.23 to 1.43)^c^	0.94 (0.8 to 1.11)
**Portuguese** *n* = 9862	1.43 (1.30 to 1.57)^c^	1.29 (0.99 to 1.68)	1.74 (1.50 to 2.02)^c^	1.40 (1.08 to 1.81)^b^
**Spanish ** *n* = 7882	0.83 (0.73 to 0.95)^c^	0.8 (0.58 to 1.10)	1.12 (0.91 to 1.38)	0.86 (0.63 to 1.20)
**French ** *n* = 4968	0.81 (0.70 to 0.95)^b^	0.65 (0.40 to 1.06)	0.83 (0.65 to 1.04)	0.68 (0.40 to 1.15)
**Polish ** *n* = 4311	1.33 (1.04 to 1.72)^b^	1.23 (0.89 to 1.71)	0.75 (0.57 to 0.99)^b^	0.77 (0.44 to 1.36)
**Somali ** *n* = 1567	0.40 (0.32 to 0.50)^c^	1.4 (0.67 to 2.86)	1.63 (1.34 to 1.98)^c^	0.87 (0.47 to 1.60)
**Other ** *n* = 19 380	0.92 (0.84 to 1.01)	1.34 (1.07 to 1.67)^c^	1.42 (1.25 to 1.60)^c^	0.84 (0.69 to 1.03)
**Not recorded** *n* = 92 676	0.98 (0.94 to 1.03)	1.07 (0.97to 1.17)	1.15 (1.08 to 1.22)^c^	1.12 (1.00 to 1.25)^b^

^a^Multiple logistic regression adjusted for age, sex, socioeconomic deprivation, ethnicity, and practice clustering. ^b^ *P*<0.05. ^c^ *P*<0.01. CHD = coronary heart disease. DM = diabetes mellitus. OR= odds ratio.

### Cardiovascular risk factors

The associations between cardiovascular risk factors and language preference groups, adjusted for the same variables as in the diagnosis analysis above, are reported in Table 3. The non-English language preference group overall had higher prevalence of obesity and smoking, and lower prevalence of raised BP. Significantly higher rates of smoking were observed in all the individual non-English language preference groups compared to the English language preference group.

**Table 3.** Association between cardiovascular risk factors at the most recent measurement and language preferences (adjusted^a^ ORs with 95% CIs).

	**Obesity **OR (95% CI)	**BP >150/90 mmHg**OR (95% CI)	**HBA1c >48 mmol/mol **OR (95% CI)	**Total cholesterol >5 mmol/l**OR (95% CI)	**Current smoker, yes or no**OR (95% CI)
**English**	1 *n* = 134 639	1*n* = 135 220	1*n* = 10 545	1*n* = 7349	1*n* = 157 841
**Non-English**	1.08 (1.04 to 1.13)^c^ *n* = 38 755	0.93 (0.88 to 0.99)^b^ *n* = 38 196	0.94 (0.76 to 1.15)*n* = 2958	1.02 (0.83 to 1.24)*n* = 2194	1.18 (1.14 to 1.21)^c^ *n* = 46 404
**Portuguese**	1.53 (1.36 to 1.73)^c^ *n* = 7785	1.12 (0.95 to 1.29)*n* = 7841	1.13 (0.88 to 1.45)*n* = 575	0.89 (0.59 to 1.33)*n* = 214	1.13 (1.02 to 1.25)^b^ *n *= 9360
**Spanish**	1.21 (1.09 to 1.34)^c^ *n* = 6304	0.65 (0.55 to 0.77)^c^ *n* = 6146	0.93 (0.65 to 1.31)*n* = 416	1.30 (0.90 to 1.90)*n* = 216	0.79 (0.7 to 0.88)^c^ *n* = 7626
**French**	0.75 (0.69 to 0.82)^c^ *n* = 4007	0.78 (0.66 to 0.93)^c^ *n* = 3887	0.73 (0.51 to 1.02)*n* = 225	0.65 (0.39 to 1.08)*n* = 132	1.43 (1.3 to 1.57)^c^ *n* = 4802
**Polish**	1.03 (0.84 to 1.26)*n* = 3392	1.72 (1.46 to 2.01)^c^ *n* = 3168	0.90 (0.50 to 1.63)*n* = 98	0.51 (0.24 to 1.08)*n* = 86	1.38 (1.25 to 1.52)^c^ *n* = 4194
**Somali**	1.12 (0.95 to 1.31)*n* = 1256	0.61 (0.50 to 0.73)^c^ *n* = 1371	1.09 (0.75 to 1.60) *n* = 135	1.8 (0.81 to 3.98)*n* = 60	1.34 (1.05 to 1.70)^b^ *n* = 1528
**Other**	0.91 (0.85 to 0.98)^c^ *n* = 16 011	0.86 (0.77 to 0.95)^c^ *n* = 15 783	1.08 (0.86 to 1.35)*n* = 1509	1.05 (0.77 to 1.43)*n* = 1516	1.31 (1.22 to 1.40)^c^ *n* = 18 894
**Not recorded**	1.14 (1.10 to 1.18)^c^ *n* = 62 116	1.05 (1.00 to 1.10)^b^ *n* = 68 872	1.13 (0.97 to 1.32)*n* = 4944	0.99 (0.86 to 1.14)*n* = 3534	1.28 (1.24 to 1.31)^c^ *n* = 80 066

^a^Multiple logistic regression adjusted for age, sex, socioeconomic deprivation, ethnicity and practice clustering. ^*b^ *P*<0.05. ^c^ *P*<0.01.

BP = blood pressure. HbA1c = glycated haemoglobin. OR = odds ratio.

### Cardiovascular monitoring

The associations between the components of cardiovascular risk monitoring and language preference groups, adjusted as before, are reported in Table 4. The non-English language preference group was significantly more likely to be monitored for smoking cessation.

**Table 4.** Association between cardiovascular monitoring recorded within a set interval and language preference (adjusted^a^ ORs with 95% confidence intervals).

	Smoking cessation history recorded within 15 months, OR (95% CI)	BP recorded within 9 months, OR (95% CI)	Cholesterol recorded within 15 months, OR (95% CI)	HbA1c recorded within 15 months, OR (95% CI)
**English** *n* = 161 938	1*n* = 60 046	1*n* = 135 228	1*n* = 7467	1*n* = 151 101
**Non-English** *n* = 47 790	1.06 (1.01 to 1.11)^b^ *n* = 16 707	0.98 (0.95 to 1.02)*n* = 38 199	1.02 (0.88 to 1.19)*n* = 2207	1.09 (0.93 to 1.28)*n* = 3047
**Portuguese** *n* = 9862	1.24 (0.96 to 1.59)*n* = 3844	1.17 (0.97 to 1.4) *n* = 7841	1.17 (0.8 to 1.71)*n* = 215	1.43 (0.91 to 2.24)*n* = 581
**Spanish** *n* = 7882	1.03 (0.83 to 1.28)*n* = 2713	0.93 (0.84 to 1.02)*n* = 6147	1.38 (0.89 to 2.13)*n* = 220	0.95 (0.66 to 1.36)*n* = 419
**French** *n* = 4968	0.87 (0.76 to 0.99)^b^ *n* = 1688	0.73 (0.67 to 0.8)^c^ *n* = 3887	0.87 (0.62 to 1.22)*n* = 133	0.92 (0.62 to 1.37)*n* = 232
**Polish** *n* = 4311	1.12 (0.76 to 1.65)*n* = 1701	0.94 (0.79 to 1.13)*n* = 3168	1.2 (0.66 to 2.18)*n* = 56	1.17 (0.56 to 2.47)*n* = 98
**Somali** *n* = 1567	1.14 (0.8 to 1.61)*n* = 443	0.95 (0.8 to 1.1)*n* = 1371	0.68 (0.38 to 1.22)*n* = 60	1.01 (0.65 to 1.57)*n* = 139
**Other** *n* = 19 380	0.98 (0.84 to 1.16)*n* = 6318	0.99 (0.91 to 1.08)*n* = 15 785	0.92 (0.73 to 1.17)*n* = 1523	1.03 (0.84 to 1.26)*n* = 1578
**Not recorded** *n* = 92 676	1.06 (1.02 to 1.11)^c^ *n* = 30 372	0.86 (0.84 to 0.89)^c^ *n* = 68 876	1.29 (1.16 to 1.42)^c^ *n* = 3584	0.98 (0.87 to 1.11)*n* = 5094

^a^Multiple logistic regression adjusted for age, sex, socioeconomic deprivation, ethnicity and practice clustering. ^b^ *P*<0.05. ^c^ *P*<0.01. BP = blood pressure. OR = odds ratio.

## Discussion

### Summary

The prevalence of CHD and DM was higher in patients with a record of non-English language preference, even after adjustment for age, sex, social deprivation, ethnicity, and practice clustering. Non-English language preference was not associated with higher prevalence of hypertension and stroke.

This study found higher rates of two cardiovascular risk factors in the non-English language preference group — obesity and smoking — which may have contributed to higher cardiovascular disease prevalence. Overall cardiovascular monitoring in patients with a preference for a language other than English was at least as good as that in the majority (English) language preference group in this inner city sample, demonstrating the effectiveness of this aspect of primary care in non-English speakers.

Some cardiovascular factors differed significantly between individual language groups, independent of ethnicity. Portuguese and Somali-speakers had higher prevalence of DM. Spanish, French and Somali-speakers had lower prevalence of hypertension.

Almost all of the patients who expressed a preference for a language other than English were first-generation immigrants born outside the UK. The cardiovascular diseases and cardiovascular risks associated with them were therefore likely to represent genetic and cultural factors of their country of origin.

Patients recorded their language preference in response to requests by their registering practice for personal health-related information. Language preference was available for 69.4% of the eligible population, although this information was not validated by English proficiency testing. Language preference may have been documented in the expectation that an interpreter would be offered. It is likely that responders expected the consulting clinician to be consulting in the majority language, English.

Cardiovascular diagnosis, risk factors, and monitoring were recorded by clinicians at a time when practice income was linked to incentive payments for this activity. Introduction of incentive targets has not been shown to remove ethnicity-related health inequalities.^5^

### Strengths and limitations

The use of census-derived ethnicity groupings may not have provided sufficient detail of ethnic background for this study. Two-thirds of patients whose preferred language was Portuguese described themselves as 'white other', a category which did not distinguish between Portuguese-speakers who were born in Brazil and those born in Portugal.

Among the strengths of this study were the large sample, the high recording level (69.4%) of language preference, the diversity of language preferences, the high proportion of patients with a non-English language preference, and the universal access to health care in the UK via the NHS. Sensitivity analysis of the group for whom language was not recorded (presented as the 'not recorded' category in Tables 1–4) suggested that differences in cardiovascular disease, monitoring, and risk factors in this group had minimal impact on the CIs of the other groups.

A limitation of this study was the use of reported preference for a non-English language as a proxy marker of low English language proficiency. There may have been patients with high proficiency in English who expressed a preference for another language. A small number of people in the area of the study may not have been registered with primary care services. This number may have included people who did not speak English and who were beyond the scope of this study.

The sample is from a setting characterised by high rates of socioeconomic deprivation and ethnic diversity. The results of this study included adjustment for the effects of both of these factors. Much of the research into ethnicity and health care focuses on non-white groups. The largest ethnic group in this non-English speaking population was ‘other white’ which, although one of the official national census categories, fails to differentiate effectively by culture, country of origin, or language. This analysis — by language preference rather than ethnicity — revealed health inequalities in cardiovascular diagnosis which are associated with risk factors specific to the non-English language preference population. This population is characterised both by deprivation and by high levels of new arrivals to the UK. Inequalities in the prevalence of cardiovascular disease were not compounded by inequalities in the recorded health care received.

The relatively small number of practices (*n* = 49) involved in this study may have concealed some clustering of population and practice characteristics not evident in this study's analysis. Also, the regression models were conducted as three separate analyses of disease prevalence, of disease monitoring, and of disease risk factors. Future analyses combining these models may provide further evidence of the role of low proficiency in the majority language as a determinant of cardiovascular disease and risk-factor prevalence.

### Comparison with existing literature

A range of adverse health outcomes for patients with low proficiency in the majority language have been shown in a US setting.^8,9^ In the UK context, an unmet need for interpreting services has been described as well as cardiovascular ethnic health inequalities.^5,6,10^ The evidence in this study is that language was not a barrier to cardiovascular monitoring in this London setting, but its use as an identifier may have highlighted inequalities in cardiovascular diagnosis and risk. While preference for a non-majority language may not be a cause of worse health care, it is likely that lack of proficiency in the majority language was associated with worse access to health information.

### Implications for research

This study reports the effectiveness of primary healthcare providers in providing equal monitoring of cardiovascular disease and risk factors for non-English language preference groups. It invites further exploration of patient factors that may contribute to increased prevalence of cardiovascular risk factors in non-majority language preference groups. These factors may relate to shared cultural factors such as smoking, exercise, and diet that qualitative analysis by language preference group might reveal. Qualitative analysis of cultural norms and health behaviours within language preference groups would support the quantitative differences set out in this study. The generalisability of this study's findings is likely to depend on the extent to which the experience of non-English language preference patients differs between areas with higher and lower levels of English language proficiency. Further research is needed to identify a broader range of healthcare needs in non-English language preference groups.
